# How to map forest structure from aircraft, one tree at a time

**DOI:** 10.1002/ece3.4089

**Published:** 2018-05-08

**Authors:** Michele Dalponte, Lorenzo Frizzera, Damiano Gianelle

**Affiliations:** ^1^ Department of Sustainable Agro‐ecosystems and Bioresources, Research and Innovation Centre Fondazione E. Mach San Michele all'Adige TN Italy

**Keywords:** aboveground biomass, airborne laser scanner, forest structure, gini coefficient, LiDAR, mapping, remote sensing

## Abstract

Forest structure is strongly related to forest ecology, and it is a key parameter to understand ecosystem processes and services. Airborne laser scanning (ALS) is becoming an important tool in environmental mapping. It is increasingly common to collect ALS data at high enough point density to recognize individual tree crowns (ITCs) allowing analyses to move beyond classical stand‐level approaches. In this study, an effective and simple method to map ITCs, and their stem diameter and aboveground biomass (AGB) is presented. ALS data were used to delineate ITCs and to extract ITCs’ height and crown diameter; then, using newly developed allometries, the ITCs’ diameter at breast height (DBH) and AGB were predicted. Gini coefficient of DBHs was also predicted and mapped aggregating ITCs predictions. Two datasets from spruce dominated temperate forests were considered: one was used to develop the allometric models, while the second was used to validate the methodology. The proposed approach provides accurate predictions of individual DBH and AGB (*R* ^2^ = .85 and .78, respectively) and of tree size distributions. The proposed method had a higher generalization ability compared to a standard area‐based method, in particular for the prediction of the Gini coefficient of DBHs. The delineation method used detected more than 50% of the trees with DBH >10 cm. The detection rate was particularly low for trees with DBH below 10 cm, but they represent a small amount of the total biomass. The Gini coefficient of the DBH distribution was predicted at plot level with *R* ^2^ = .46. The approach described in this work, easy applicable in different forested areas, is an important development of the traditional area‐based remote sensing tools and can be applied for more detailed analysis of forest ecology and dynamics.

## 1 INTRODUCTION

Forest structure influences both the carbon content of forests and its changes in time (Fischer et al., 2016). Forest structural diversity can be described both in terms of horizontal and vertical distribution of forest parameters such as species, leaf area, tree diameter, crown size (Hubbell, Ahumada, Condit, & Foster, 2001) and can be considered one of the surrogates of biodiversity (Pach & Podlaski, 2015). Traditional methods for assessing forest structure comprise field inventories and optical remote sensing (i.e., aerial photography, stereoscopy). Airborne laser scanning (ALS) is a commonly used tool in forest ecology and environmental mapping (Vierling, Vierling, Gould, Martinuzzi, & Clawges, 2008), most often providing stand‐level estimate of forest variables (Næsset, 2002, 2004). However, ALS datasets are increasingly collected at high enough point density to study forests at individual tree level (Coomes et al., 2017; Dalponte & Coomes, 2016) with the potential to impact on the planning of silvicultural activities and cutting regimes, but also to describe forest parameters in details.

In a worldwide study, Jucker et al. (2017) produced allometric equations by which tree diameter at breast height (DBH) and aboveground biomass (AGB) could be predicted from height and crown diameter measurements. These two tree attributes can be predicted with ALS data as they are the standard output of almost every individual tree crown (ITC) delineation method (Zhen, Quackenbush, & Zhang, 2016). This opens at the possibility to map forests over large areas from ALS, with the sort of detail only previously possible by intensive field work. So recent developments in sensors, estimation equations, and big data processing are providing new opportunities to implement of individual tree‐based models over entire states (Shugart et al., 2015). Single trees maps permit to analyze forest structure at different scales and many important forest functions, such as tree diversity—carbon storage relationships are scale dependent (Sullivan et al., 2017).

In this work, we show a simple and effective method to map at ITC level, diameters, and biomass using height and crown diameters measured from ALS data. To understand whether our results can be generalized at least in forest ecosystems with similar characteristics, we developed estimation equations in one area and then we applied them in a second one. A comparison with a standard area based method is also provided.

We also predict and map the Gini coefficient (Gini, 1921), a concise indicator to assess the structural diversity of forest stands and considered one of the most suitable indicator for studying the complexity of forests (Valbuena, Packalén, Martı′n‐Fernández, & Maltamo, 2012), to demonstrate the power of ALS data in the measurements of forest structure. Gini coefficient can be used both as an indicator to assess tree competition and succession and to evaluate the effect of human management that strongly impact tree diameter distribution (Valbuena, Eerikäinen, Packalen, & Maltamo, 2016).

## 2 MATERIALS AND METHODS

### 2.1 Field and ALS data

Two datasets were used in this study. The first dataset (Paneveggio) was used only to develop the DBH and AGB models. The Paneveggio study area (about 4 km^2^) is located in the Italian Alps (46°17′48″ N 11°45′26″ E). The main species of the forest is Norway spruce (*Picea abies* (L.) H. Karst) with the presence of some minority species, such as European larch (*Larix decidua* Mill.), green alder (*Alnus viridis* (Chaix.) D.C.), and Swiss pine (*Pinus cembra* L.). The field data consists of 47 circular sample plots of different radii (two plots of 4 m, one of 6 m, two of 7 m, 12 of 13 m, and 30 of 20 m). Within each sample plot, tree species (90% Norway spruce, 3% European larch, 3% green alder, 1% Swiss pine, 3% other species), DBH (min. 4 cm; max. 109 cm; mean 27 cm), height (min. 1.6 m; max. 45.7 m; mean 19.3 m), and tree coordinates were recorded for all trees with DBH > 4 cm. For 1,182 trees, also the crown diameters (min. 0.5 m; max. 17.7 m; mean 4 m) were measured.

The second dataset (Pellizzano) was used to validate the models. The Pellizzano study area (about 32 km^2^) is also located in the Italian Alps (46°17′49″ N 10°46′17″ E). The dominant tree species are *P. abies* (L.) Karst., with the presence of other coniferous species (e.g., *Abies alba* Mill., *L. decidua* Mill., *P. cembra* L., *Pinus sylvestris* L., and *Pinus nigra* J.F. Arnold) and broadleaves species (e.g., *Populus tremula* L., *Betula* spp.). Further details about the study area can be found in Dalponte and Coomes (2016). The field data were collected on 47 circular sample plots 15 m radius. Within each sample plot, tree species, DBH (min. 4 cm; max. 89 cm; mean 28.8 cm), height (min. 1.9 m; max. 42.6 m; mean 19.8 m), and tree coordinates were recorded for all trees with DBH > 4 cm. A total of 1,952 trees were recorded. ALS data were acquired using a Riegl LMS‐Q680i sensor system operating with a pulse repetition frequency of 400 kHz. Up to four echoes per pulse were recorded, and the resulting pulse density was about 48 m^−2^.

Aboveground biomass for the field trees of the two datasets (Paneveggio: min. 1 kg, max. 4,086 kg, mean 406 kg; Pellizzano: min. 1 kg, max. 3,250 kg, mean 512 kg) was predicted using the models of Scrinzi, Galvagni, and Marzullo (2010) and field measured height, DBH, and species.

### 2.2 Methods

#### 2.2.1 Proposed method

**Figure 1:**
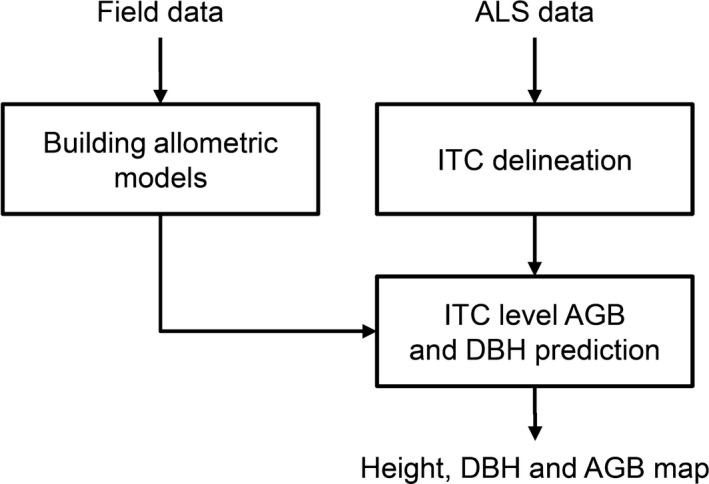
**Architecture of the proposed methodology**

In Figure 1, the architecture of the proposed methodology is showed. First of all, using a set of field measured trees (in our case the Paneveggio dataset), following the methodology of Dalponte and Coomes (2016) and Jucker et al. (2017), allometric models relating DBH and AGB to height and crown diameter are developed. The models were in the form:

(1) $$\text{DBH}=a\;\times \;\left(H^{b}\right)\;\times \;\left(1+c\;\times \;\text{CD}\right)$$

(2) $$\text{AGB}=a\;\times \;{\left(H\;\times \;\text{CD}\right)}^{b}$$

where DBH is the diameter at breast height in cm, AGB is the aboveground biomass in kg, *H* the height in meters, and CD the crown diameter in meters. The models are parametrized using the *nlrq* function of quantile regression package *quantreg* in R (τ = 0.5) which is less sensitive to heteroscedasticity than conventional least‐square regression (Koenker & Park, 1996).

Secondly, ITCs are delineated on the ALS dataset (in our case the Pellizzano dataset). Many ITC delineation for ALS data methods exist, and in this work, we used the delineation algorithm of the R package *itcSegment*. This ITC delineation approach finds local maxima within a rasterized CHM, designates these as tree tops and then uses a decision tree method to grow individual crowns around the local maxima. The output of the delineation method is the detected trees with the information related the height (95th percentile of the ALS points inside the delineated crown) and the crown diameter. For additional details on the delineation method, see the supplementary material of Dalponte and Coomes (2016).

Diameter at breast height and AGB are predicted for each ITC using the models in Equations 1 and 2 and height and crown diameter extracted from ALS data. Gini coefficient of the DBHs (GiniDBHs) was computed in plots with at least 10 trees for a total of 43 plots using field data and ITC predicted DBHs.

#### 2.2.2 Area‐based method

In order to have a comparison with a standard approach, models for AGB and the Gini coefficient of DBHs prediction at plot level were also developed based on Paneveggio dataset using the following formulation:

(3) $$\text{AGB}=a\;\times \;{\text{TCH}}^{b}$$

(4) $$\text{GiniDBHs}=a\;\times \;{\text{TCH}}^{b}$$

where TCH is the top‐of‐the‐canopy height metric (Asner & Mascaro, 2014) computed for each plot, AGB is the aboveground biomass of each plot, and GiniDBHs is the Gini coefficient of DBHs for each plot. We chose simple models in order to have a higher generalization ability, and in order to have an approach comparable to the one proposed in section 2.2.1. It is worth noting that in this case, differently than for the proposed method, ALS data are necessary also to build the model.

#### 2.2.3 Validation procedure

To evaluate the models of Equations 1–4, the root mean square error (RMSE), average mean difference (¯$\overset{¯}{D}$), and coefficient of determination (*R* ^2^) were calculated on both the Paneveggio and Pellizzano datasets. Regarding the Paneveggio dataset, a 10% cross‐validation procedure was performed using 90% of the samples (i.e., field measured trees for the proposed method, and field plots for the area‐based method) to build the model and 10% to evaluate it. The procedure was repeated 100 times. The RMSE, $\overset{¯}{D}$, and *R* ^2^ values are the mean values over all the repetitions. This procedure was used only to have an evaluation of the models based on field data. Regarding the Pellizzano dataset RMSE, $\overset{¯}{D}$, and *R* ^2^ were computed between field and predicted values of DBH and AGB of the ITCs that were matching a field tree, and of the AGB and GiniDBHs of the plots. The delineated ITCs were automatically matched to the trees measured in the field. If only one field measured tree was included inside an ITC, then that tree was associated to that ITC. In the case of more than one field measured tree was included in a segmented ITC, the field measured tree with the closer height to the ITC height was chosen (Dalponte & Coomes, 2016).

## 3 RESULTS

### 3.1 Proposed method

**Figure 2:**
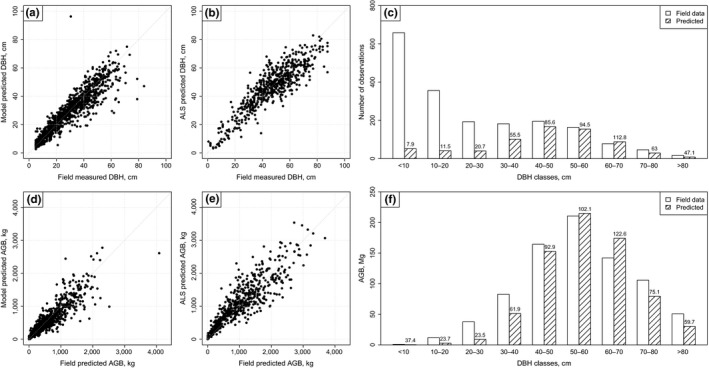
**(a) DBH measured in the field versus model predicted DBH (Equation 1) for the field trees of Paneveggio; (b) DBH measured in the field versus model predicted** **DBH** **on the ITCs matched with field trees of Pellizzano dataset; (c) DBH class distribution of the field measured and ALS detected trees of Pellizzano dataset, along with the percentage of detected trees for each** **DBH** **class; (d) field predicted AGB versus model predicted** **AGB** **(Equation 2) on the field data of the Paneveggio dataset; (e) field predicted** **AGB** **versus model predicted** **AGB** **on the** **ITCs** **matched with field trees of Pellizzano dataset; (f) DBH class distribution of the** **AGB** **of the field measured and** **ALS** **detected trees of Pellizzano dataset, along with the percentage of detected** **AGB** **for each** **DBH** **class**

The individual accuracies of the models on the field data are high (Equation 1: *R* ^2^ = .85, RMSE = 6.43, $\overset{¯}{D}$ = 0.01; Equation 2: *R* ^2^ = .78, RMSE = 226.63, $\overset{¯}{D}$ = 4.02), and this is confirmed also by the scatterplots in Figure 2a–d. ITC level results for DBH and AGB prediction are showed in Figure 2b–e. Accuracies are lower compared to the ones of the models but the systematic error is really small (Equation 1: *R* ^2^ = .78, RMSE = 8.10, $\overset{¯}{D}$ = 0.06; Equation 2: *R* ^2^ = .78, RMSE = 345.64, $\overset{¯}{D}$ = 0.47). The detection rate of the delineation method on the Pellizzano dataset considering all the field measured trees in the 47 plots was 30.6% with a the commission error of 8.3%, and an accuracy index of 22.3%. The delineation method used detected more than 50% of the trees with DBH >10 cm (see Figure 2c). Only about 8% of the trees with DBH <10 cm were detected. The detection rate was particularly low for trees with DBH below 10 cm, but they represent a small amount of the total biomass (Figure 2f). For the trees with DBH above 30 cm, we registered the highest detection rates (>80% in total) with also the highest percentage of detected AGB.

**Figure 3:**
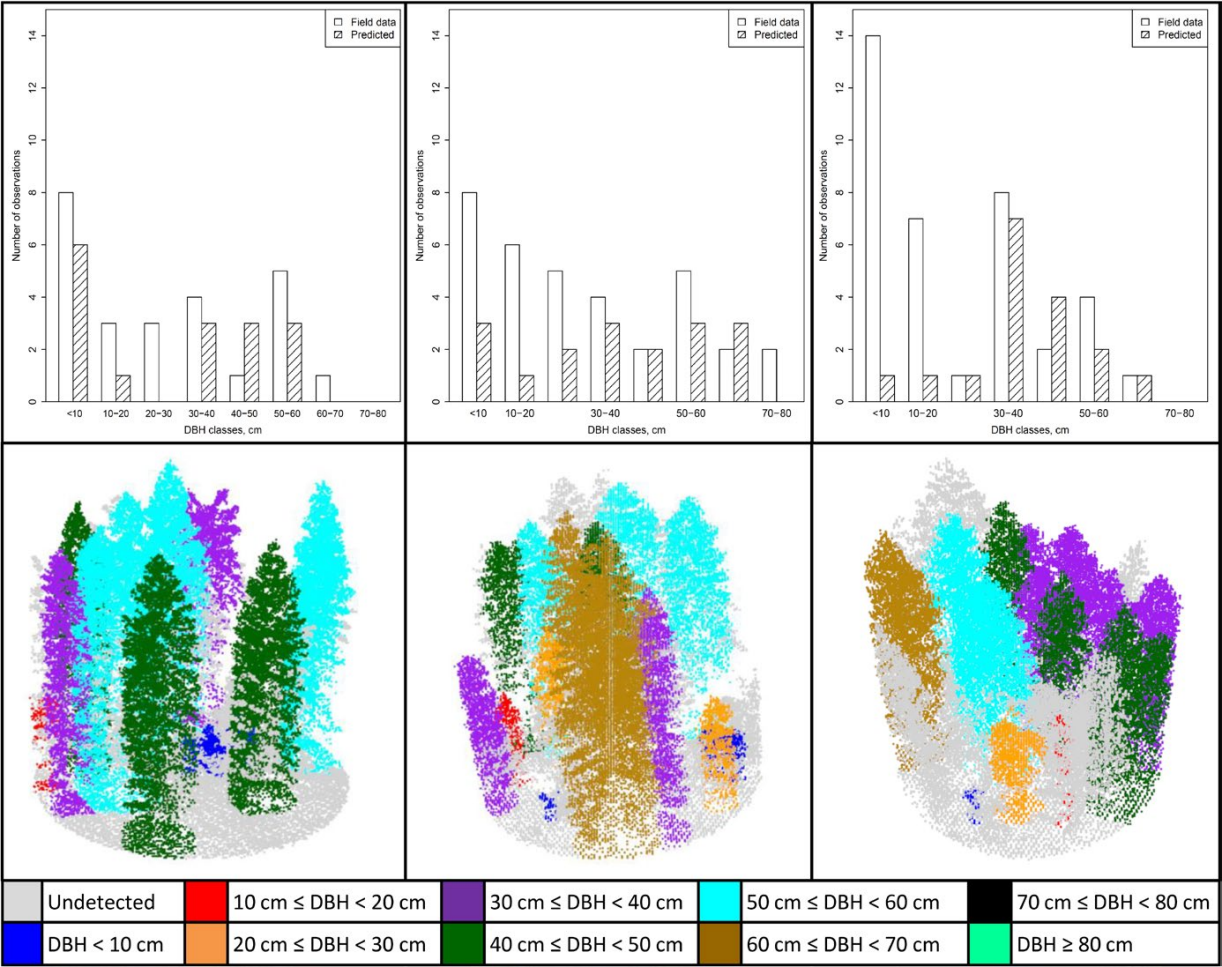
**Example of ALS data over three plots and the detection rate for each DBH class in the three plots considered. The colors of the** **ALS** **points are related to the DBH class predicted. Gray points represent points not associated to any ITC**

Figure 3 shows an example of tree detection on three plots. Big trees are generally detected and the DBH is correctly predicted, even if some error happens for trees diameter close to the border among two classes. Small trees are detected only if they are not dominated by other trees and where canopy density is low (Figure 3 left panel). The presence of young small trees in the understory of dense forest is difficult to detect with ALS data (Figure 3 right panel).

The Gini coefficient of the DBH distribution was predicted at plot level (700 m^2^ plots) using the predicted ITC's DBH. The results were *R* ^2^ = .46, RMSE = 0.21, $\overset{¯}{D}$ = −0.36, The slope of the linear relationship among field and predicted Gini coefficient was 0.5.

### 3.2 Area based method

**Figure 4:**
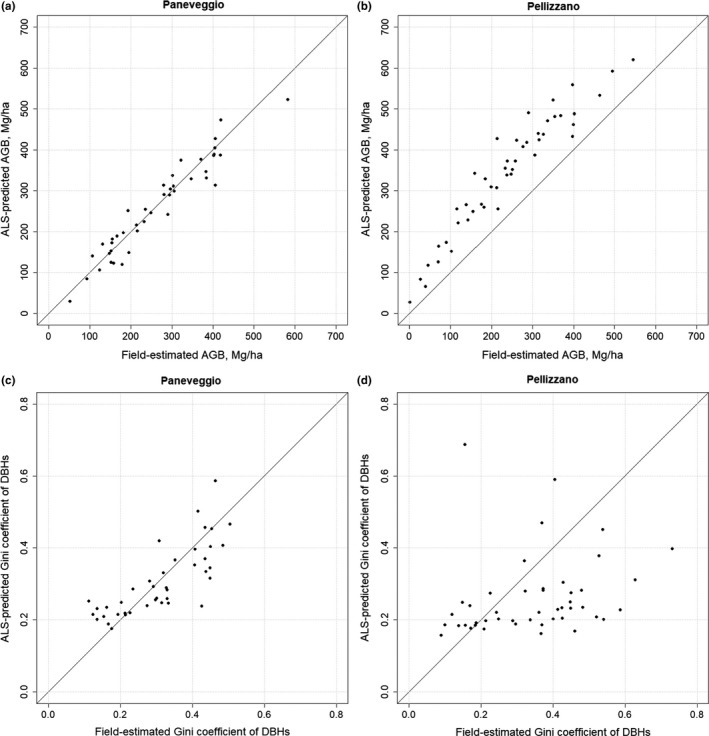
**Field‐estimated versus ALS‐predicted AGB (a–b) and Gini** **DBH** **s (c–d) at plot level on the two datasets considered**

The individual accuracies of the models on the Paneveggio data set are high (Equation 3: *R* ^2^ = .91, RMSE = 32.3, $\overset{¯}{D}$ = 26.8; Equation 4: *R* ^2^ = .65, RMSE = 0.08, $\overset{¯}{D}$ = 0.07), and this is confirmed also by the scatterplots in Figure 4a–c. AGB and GiniDBHs prediction on Pellizzano dataset are showed in Figure 4b–d. Accuracies are lower compared to the ones of the models (Equation 3: *R* ^2^ = .91, RMSE = 113, $\overset{¯}{D}$ = 104; Equation 4: *R* ^2^ = .01, RMSE = 0.25, $\overset{¯}{D}$ = 0.17). The RMSE, and $\overset{¯}{D}$ for the AGB prediction are quite high, while the prediction of GiniDBHs on the Pellizzano dataset is very inaccurate.

## 4 DISCUSSION

The results showed that using only the ALS information over an area and equations developed for a certain type of forest (i.e., spruce dominated temperate forests), it is possible to map accurately the DBH and AGB at ITC level, in other forests with similar characteristics. This is an important result because it demonstrates that using allometries developed using only local field data at a certain time, without the use of ALS data, we can predict ITC level attributes and forest structure over an extensive region covered with ALS data at any other time. The ITC approach was demonstrated to be very useful for many applications in which data on minority species are important, as the ITC approach allows detection of the AGB distributed by species and class diameters (Kandare, Dalponte, Ørka, Frizzera, & Næsset, 2017). The possibility to detect single ITCs and the attributes associated with them is useful in the context of individual‐based models, a new generation of models that integrate individual‐level mechanisms with ecosystems ecology and functionality (Grimm, Ayllón, & Railsback, 2017). The main strength of the proposed approach is that, once the allometries have been developed, just using ALS data it is possible to obtain height, DBH and AGB for each detected ITC without needing any field data, helping for a more precise forest management without extra costs in large areas. One issue that may rise is the heteroscedasticity of the prediction error for AGB and DBH at ITC level. This is due to the fact that the relationship among DBH (or AGB) and CD and *H* changes with tree age: after a certain age trees grow less (or nothing) in height, but they can still grow in DBH. This problem can be solved developing models that can change according to the tree height, but at the price of having more complex (and probably less general) models.

One of the main unresolved problems is that ITC delineation methods fail at detecting suppressed and understory trees (Figure 2c). The tendency of missing trees will increase in multilayer and dense forests. This is the case of some of the plots of the Pellizzano dataset. In a previous comparative study of delineation methods and forest types in the Alps, Eysn et al. (2015) showed that the plots of Pellizzano were the most complex among the ones analyzed. In one‐layer forests, usually it is possible to obtain higher detection rates, even over 70% (Eysn et al., 2015; Vauhkonen et al., 2011). The detection error creates a bias in the AGB estimations at plot and stand level. In Dalponte and Coomes (2016), a correction factor is proposed to eliminate this bias. This approach can be effective, and solve the problem, but with the limitation that the correction factor is not universal and it needs to be determined for each dataset, and for each delineation method used. In the literature, some methods have been proposed to improve the detection of suppressed and understory trees, but they are still experimental (e.g., Ferraz et al., 2015). In the future, we may have operational methods that can detect dominated trees, including the use of UAV ALS data that can provide datasets with an extremely high point density and they can be used for a wide variety of ecological applications (Anderson & Gaston, 2013; Christie, Gilbert, Brown, Hatfield, & Hanson, 2016). The use of terrestrial laser scanning can help also in having a very detailed characterization of the forest, including suppressed trees, but it can be used only on small areas, as it is not feasible its use over large areas.

Many studies can be found in the literature on area‐based methods for the prediction of AGB (e.g., Asner & Mascaro, 2014; Gizachew et al., 2016; Vaglio Laurin et al., 2014), and some studies compare area‐based methods with ITC methods (e.g., Coomes et al., 2017; Kandare et al., 2017). If the interest of the end user is in total AGB at plot level usually area‐based methods provide better results than ITC methods, and both Coomes et al. and Kandare et al. showed it. This is due to the fact that the developed models are site specific and they are not biased by the omission error of ITC delineation methods. Despite this we think that some drawbacks exist: (1) in area‐based methods the prediction of AGB per species or per DBH classes is less accurate, or in some cases impossible (Kandare et al., 2017); and (2) area‐based methods need field data collected all in the same way. Going into details from our results emerged that plots should be located exactly in the area that needs to be analyzed, as area‐based models used out of the dataset where they are developed are quite inaccurate (e.g., AGB model, Figure 4b), or even useless (e.g., GiniDBHs model, Figure 4). Additionally in area‐based methods, all the plots need to be of the same size, and the pixel size of the final map is constrained by the plot size (e.g., using field plots of 1 ha size we can generate an AGB map of 100 m spatial resolution). Additionally according to the sampling strategy adopted, the final results may be different (Gregoire & Valentine, 2008). Differently the proposed approach uses field data not specifically collected in the analyzed area, as they do not need to be matched with the ALS data. Additionally they can be acquired in different time periods, and without being grouped in plots, simplifying a lot the field data collection. It is worth noting that the proposed approach does not involve the use of tree positions.

Individual tree crown level data permit to describe forest structure and can support many forest ecology applications that need detailed spatial description of the studied area. This is showed by the results on the distribution of AGB per DBH class and on the prediction of the Gini coefficient. Gini coefficient was demonstrated to be useful to describe the peculiarities of the boreal forests managed with different practices (Valbuena et al., 2016). The possibility to extract structural indices in large areas is useful also for the improvement of forest management, considering that these indices are correlated with forest productivity (Bourdier et al., 2016; Zhang & Chen, 2015). The effect of structural diversity on growth and forest dynamics needs more attention (Forrester & Bauhus, 2016), and we need to link efficiently tree‐ and stand‐level patterns. ITCs and structural indices, predict in large areas, could help to close this gap.

Airborne laser scanning data characteristics necessary to apply the proposed approach needs to have a point density above a certain number. A previous study showed that with a point density above 5 pts/m^2^, it is already possible to have results similar to the ones obtained with much higher point densities (Kandare, Ørka, Chan, & Dalponte, 2016). In the past, many operational flights had a quite low point density (one or less points per square meter), but it is expected the future acquisitions will be with 5 or more points per square meter allowing the use of ITC methods.

The approach described in this work, based on ITC detection, easy applicable in different forested areas, is an important development of the traditional area‐based remote sensing tools and can be applied for more detailed analysis of forest ecology and dynamics. A further step will be the use of ALS and hyperspectral data together to identify the species at tree level and to prepare a complete map of forest structure, biodiversity, and biochemical properties that could be measured with spectral reflectance. It is necessary to underline that hyperspectral data are much more complex to process than ALS data. Moreover, there are much less availability of these data over wide areas, while ALS data are widely available, for example, many European countries have full coverage with ALS data.

## CONFLICT OF INTEREST

None declared.

## AUTHORS CONTRIBUTION

M.D. and D.G. conceived and designed the experiments; M.D. performed the experiments and analyzed the data; L.F collected and processed the field data; M.D. and D.G. wrote the manuscript; M.D., L.F., and D.G. revised the manuscript before submission.
